# Constrained Peptides as Miniature Protein Structures

**DOI:** 10.5402/2012/692190

**Published:** 2012-09-26

**Authors:** Hang Yin

**Affiliations:** Department of Chemistry and Biochemistry and BioFrontiers Institute, University of Colorado at Boulder, 596 University of Colorado at Boulder, Boulder, CO 80309-0596, USA

## Abstract

This paper discusses the recent developments of protein engineering using both covalent and noncovalent bonds to constrain peptides, forcing them into designed protein secondary structures. These constrained peptides subsequently can be used as peptidomimetics for biological functions such as regulations of protein-protein interactions.

## 1. Stabilized and Destabilized **α**-Helices 

*α*-Helices have been found to be the secondary structure about 40% of all residues in natural proteins adopt [1], and they are widely used as fundamental recognition elements in many naturally occurring protein complexes, such as Bcl-2/Bak, MDM2/p53, calmodulin/smooth-muscle-myosin-light-chain kinase, Vav/DH domain, and CREB/CBP [2–5].

**Figure 1:**
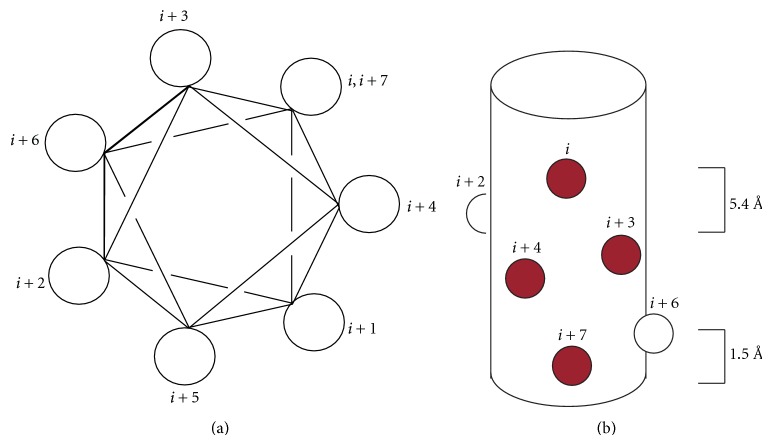
The structure of an *α*-helix. (a) The helical wheel diagram. (b) Surface displacement of residues on an *α*-helix surface.

A typical *α*-helix completes one rotation with 3.6 amino acid residues, in which each has backbone dihedral angles of Ψ = −41° and Φ = −62° [6]. This results in the helix having a rise of 1.5 Å/residue or 5.4 Å/turn [7]. Therefore, the side chain of a certain residue at the position *i* projects from the same face with the side chains at the *i* + 4 and the *i* + 7 positions in the sequence (Figure 1). The backbone of the *α*-helix is primarily stabilized by hydrogen bonds between the carbonyl of residues *i* and the carboamide of residues *i* + 4, which all point in the same direction [6]. Because the hydrogen bonding sites on the first and last turns of an *α*-helix are unfulfilled, a macrodipole is produced [8, 9]. The positive end of the dipole is centered at the N-terminus and the negative at the C-terminus. The total dipole is augmented if the peptide existed in conditions where both the termini are ionized.

It can be imagined that isolated helical peptides would be ideal inhibitors of macromolecular interactions [10]. However, because many peptides, especially those with less than ten residues, rarely contain sizable degrees of helicity in isolation, much work has been done toward the goal of helix induction and stabilization [11]. The goal of this paper is to highlight the chemical strategies employed to stabilize protein secondary structures and the applications of the constrained peptides in regulating protein-protein interactions. 

### 1.1. Covalent Stabilization

**Figure 2:**
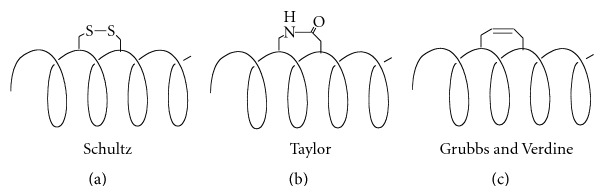
Examples of using covalent linkages to stabilize *α*-helices.

**Figure 3:**
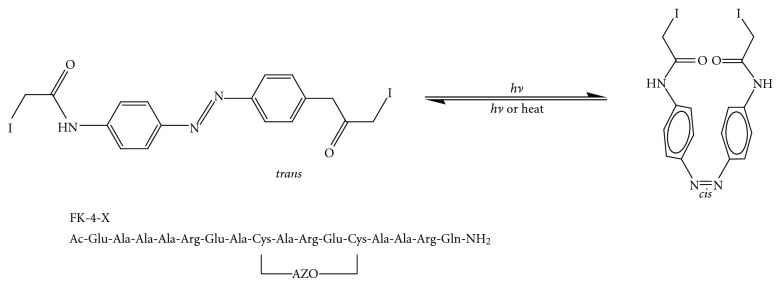
Structure of azobenzene reagents and the primary sequence of the cross-linked peptide.

The formation of covalent linkages between adjacent residues in peptides has been shown to impart stabilization to the helical form of the peptide. Disulfide bonds, lactam linkages, hydrazones, and carbon-carbon bonds have all been used to link *i* to *i* + 4 or *i* + 7 residues in a peptide and promote helicity (Figure 2). The particularly noteworthy examples include the creative approach Jackson et al. have conducted, using a redox-triggered disulfide bond to influence the helix-coil transition [12] and the medicinally useful stabilization techniques of lactam tethers developed by Taylor et al. [13, 14]. Both disulfide bonds and lactam linkages could induce the conserved pentapeptide motif (LXXLL) to adopt an *α*-helical structure [15, 16]. Fairlie and coworkers have shown their efforts on downsizing protein to short synthetic peptides with strategically lactam bridged *α*-helical structures that are stable in water. Their work covered viral (respiratory syncytial virus (RSV) F protein, HIV rev), bacterial (*Streptococcus pneumonia* competence stimulating peptide (CSP)), and human (nociceptin) proteins [17, 18]. Flint et al. have shown that linkage of a diazobenzene unit through two cysteine thiols can be used to both stabilize and destabilize the helical form of a peptide under photocontrol [19]. Linkage of the photoaddressable unit azobenzene (AZO) to the *i* and *i* + 4 residues of the helix allows stabilization of the helix in the *cis-*form, whilst linkage between the *i* and *i* + 11 results in destabilization on *trans/cis*-isomerization (Figure 3). The results are attributed to the shorter distance between the aromatic units of the *cis*-isomer as compared to the *trans* isomer. 

Blackwell and Grubbs have demonstrated that linkage through olefin metathesis of two O-allyl serines at *i* and *i* + 4 positions can facilitate the transition from an *α*-helix to a 3_10_ helix in nonpolar solvents [20]. These preliminary results offered great promise due to the advent of water-soluble metathesis catalysts and ready functionalization of serine residues. In a more recent example, Schafmeister et al. have elaborated upon this work to show that enhanced and compromised helix stability can be conferred upon the C-terminal peptide of RNase A via incorporation and subsequent metathesis of *α*,*α*-disubstituted amino acids [21]. Linkage of *i* and *i* + 7 residues with an 11-carbon linker increased the helicity from 40% to 66%, whereas linkage with a 9 or 10 carbon linker decreased helicity by 21% and 12%, respectively. Linkage with a 12-carbon linker conferred only a small increase in helicity. Trypsin proteolysis experiments demonstrated that the peptides modified at the *i* and *i* + 7 residues with an 11-carbon linker had increased proteolytic stability as the rate of cleavage was decreased 41-fold upon olefin metathesis. The Verdine group further elaborated this “peptide stapling” strategy in which an all-hydrocarbon cross-link is generated within natural peptides by ruthenium-catalyzed olefin metathesis of inserted *α*,*α*-disubstituted nonproteogenic amino acids bearing olefinic side chains, and use it in some antitumor fields [22]. They utilized hydrocarbon-stapled helices to target the intracellular protein-protein interaction between the activation domain of p53 and its negative regulator HDM2, resulting in upregulated expression of p53 and activation of apoptotic signal [23]. This group also found that hydrocarbon-stapled helices could inhibit coactivator mastermind-like (MAML) protein 1 binding to transcription factors CSL-Notch (ICN), and suppress the expression of Notch-mediated genes [24].

Patgiri and coworkers have developed another strategy for the stabilization of *α*-helices involving replacement of one of the main chain intramolecular hydrogen bonds with a covalent linkage [25]. The transition theory suggests that energetically demanding organization of three consecutive amino acids into the helical orientation limits the stability of short *α*-helices [26, 27]. Replacement of the N-terminal backbone hydrogen bond between the *i* and *i* + 4 residues with a covalent carbon bond through a ring-closing metathesis reaction drives the formation of an *α*-turn [28]. The hydrogen bond surrogate (HBS) strategy induces preorganized *α*-turns to overcome the intrinsic nucleation barrier and initiate helix formation [29]. HBS helices have been demonstrated to target with high affinity in cell-free and cell culture assays [30, 31].

### 1.2. Noncovalent Stabilization

**Figure 4:**
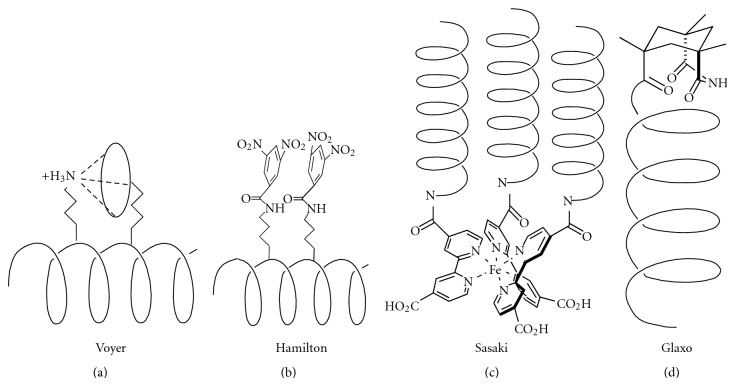
Examples of helical stabilization with noncovalent interactions.

Stabilization of *α*-helical structure has also been achieved using noncovalent interactions between appropriately spaced residues in a peptide chain. Notably metal-ligand interactions, designed host-guest interactions, salt bridges, cation-*π* interactions, and *π*-*π* stacking have resulted in helix stabilization (Figure 4). A report by Kelso and coworkers has shown that such metal-ligand interactions can be used to stabilize the helical form of even the shortest peptides [32]. AcHAAAHNH_2_ ligates to the vacant *cis-*coordination sites of Pd(en)(NO_3_)_2_ and confers *α*-helical stability as shown by 2D ROESY in DMF and water. This strategy has recently been extended further to design 5, 10, and 15 residue nonhelical peptides that correspond to the Zn^2+^-binding *α*-helix active site of thermolysin [33]. Metal ion coordination of Pd(en)^2+^ to histidine residues spaced three residues apart, that is, *i* and *i* + 4 result in formation of a 22 membered macrocycle that has a significant population of an *α*-helical conformation in DMF and water [34]. Phosphine-ruthenium coordination has also served to stabilize helical conformations in a similar manner. Shinkai et al. used a technique developed by Ruan et al. to bridge the backbone on one face of a helix and stabilized the helical structure of the S-peptide of ribonuclease S to effectively turn on and off enzymatic activities in the presence of various metals [1, 35, 36]. Ghadiri et al. and Lieberman and Sasaki both designed helical bundles that were formed in the presence of coordinating metals [37–39]. Another example has been reported by Huang and coworkers who employ an amidolinked BipyRu^II^(Bipy)_2_ which on oxidation to Ru^III^ causes increased polarization of the amide causing a transition from a random coil to *α*-helical conformation [40]. Cline and coworkers utilize model *α*-helical peptides containing two cysteine residues in various sequential arrangements and spatial locations to study the structural effects of arsenic binding. With *i* and *i* + 1, *i* + 2, or *i* + 3 arrangements, CD spectroscopy shows that As(III) coordination caused helical destabilization when Cys residues are located at central or C-terminal regions of the helix. In contrast, helical stabilization was observed for peptides containing *i*, *i* + 4 Cys residues [41]. 

Host-guest interactions have recently been employed to develop conformationally sensitive sensors by Matsumura and coworkers [42]. The peptide EK6R employing a *β*-cyclodextrin (CD) and dansyl group (Dns) at the eighth and fifteenth positions was shown to exhibit enhanced helicity as a result of the formation of an inclusion complex between the CD and hydrophobic Dns group. Competition for the CD cavity with various guests causes a decrease in Dns fluorescence resulting from the transition to aqueous media. GdnHCl-induced denaturation of the peptide demonstrated the importance of the correct presentation of CD and Dns along the surface of the peptide scaffold in order for effective sensing. Voyer and Guérin demonstrated that peptides incorporating a crown ether-containing amino acid could stabilize a helix by binding to another residue with an ammonium functionality [43]. Wilson et al. reported cyclodextrin dimmers as the helical template. The chelated binding of cyclodextrin-based receptor with the two hydrophobic side chains in the *i*, *i* + 11 positions of an oligopeptide was shown to be able to induce helicity in the peptide. CD experiments revealed that a dimeric *β*-cyclodextrin receptor synthesized from a [1,1′-biphenyl]-4.4′-dithiol core demonstrated an ability to fold a designed peptide bearing the artificial amino acid L-p-t-butylphenylalanine in the *i*, *i* + 11 positions [44]. 

The role of cation-*π* interaction in protein secondary structure stabilization has not been thoroughly studied until lately. Some early studies have suggested that cation-*π* interactions contribute more in stabilization than salt bridges on a solvent-exposed protein surface [45]. Kallenback et al. demonstrated that the Trp-Arg (*i*, *i* + 4) pair stabilized the *α*-helical conformation with a quantified stabilization energy of –0.4 kcal/mol [46, 47]. Gallivan and Dougherty conducted computational simulation to provide energetic evaluations to all potential cation-*π* interactions in a protein [48]. It was shown that the side chain of Arg is more likely than that of Lys to be in a cation-*π* interaction. However, Tsou and coworkers reported the stabilization of *α*-helices obtained by Phe-Lys cation-*π* interaction in water with a stabilizing energy of –0.4 kcal/mol, which is comparable to the Arg-Trp pair [49]. Rather than a “face to face” orientation, the Arg-Trp pair takes the Phe-Lys pair adopt through a “point to face” mode due to the lack of delocalization of the positive charge on the side chain. Three different arm lengths of the ammonium-containing side chains were screened (Lys, ornithine, and diamniobutanoic acid residues). Orn (*i*) was identified to have the optimal length in building interaction with Phe at the *i* + 4 position, indicating that the subtle factors, such as side chain length, influence the interaction energies in designed systems.

**Figure 5:**
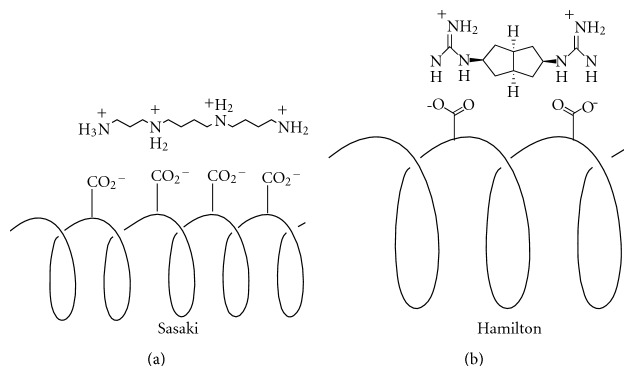
Examples of helical stabilization through intermolecular interactions.

A logical progression from the stabilization of peptides through intramolecular interactions is the stabilization of peptides through intermolecular interactions (Figure 5). Albert et al. have shown in several studies that the *α*-helical form of a peptide can be stabilized by the interaction of *bis*-guanidinium receptors with aspartate residues at the *i* and *i* + 4 residues on a peptide [50]. An alternative approach to stabilize a helix was accomplished through the use of *π*-*π* stacking interactions [51]. Similarly Tabet and coworkers have shown that naturally occurring spermine can stabilize the *α*-helical form of a peptide by binding to aspartate residues at the *i*, *i* + 4, *i* + 7, and *i* + 11 residues [52]. This effect was capitalized on by Hamachi et al. to stabilize ribonuclease S-peptide [53]. Related studies in the Hamilton laboratory have shown that binding of the tetraguanidinium receptor 1 to an appropriately designed tetra-aspartate peptide at *i*, *i* + 3, *i* + 6, and *i* + 9 positions can result in a stabilization of the *α*-helical form of the peptide [54, 55]. In an elegant extension of this work, the design of a “molecular hinge” was exploited (Figure 5) [56]. The target peptides incorporate four aspartate groups, each flanked above or below by aromatic residues on the helix surface. In this way, the tetraguanidinium receptor 1 can make both hydrogen bonds and cation-*π* interactions with side chain groups on the peptide. It has been shown that the peptide receptor 1 not only binds with high affinity but also acts to strongly stabilize the helix conformation of the peptide.

**Figure 6:**
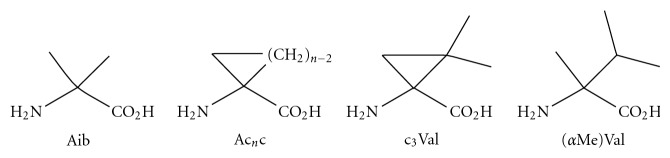
Examples of the Ca-tetrasubstituted *α*-amino acids.

The strategy to use a C*α*-tetrasubstituted amino acid to constrain peptide to form helices was employed by several groups recently. Schievano et al. reported that Aib-rich (Figure 6) peptides containing lactam-bridged side chain adopted a right-handed 3_10_ helix conformation [57]. The 3_10_ helices are another important class of secondary structural element in proteins. Traditionally, it is a challenge to identify 3_10_ conformations because of their similar spectroscopic characteristics to *α*-helices. The backbone dihedral angels *ϕ* and Ψ of the two helices differ only by 6° and 12°, respectively [58]. Peggion and coworkers managed to analyze the constrained peptide by conducting a detailed CD and NMR analysis followed by a NOE-distance-based structure ensemble calculations using the XPLOR simulated annealing protocol. They used an *i*, *i* + 3 lactam bridge, which was both 3_10_ helix stabilizing and *α*-helix destabilizing, to assure the constrained peptide predominantly fold into the 3_10_ helix conformation. It was found the Aib-rich peptide oligomers tend to form 3_10_ helix upon chain elongation. The presence of the 3_10_ helices was further supported by the temperature coefficients of the –NH proton chemical shifts, which are in the range usually observed in an H-bonded structure. Similarly, the employment of (R)-c_3_Val(1-amino-2,2-dimethylcyclopropane-1-carboxylic acid), an extremely strained analogue of the previously used (*α*Me)Val (Figure 6), has been reported [58, 59]. It was shown that c_3_Val was a good *β*-bend and helix former though not as efficient as (*α*Me)Val. ^1^H-NMR experiments showed in CDCl_3_ solution of the c_3_Val constrained peptide that the N_93_ H to N_7_ H protons are intramolecularly H-bonded, suggesting that the secondary structure the peptide adopted in CDCl_3_ is a 3_10_ helix. The conclusion was further confirmed by the X-ray crystal structure.

### 1.3. Other Stabilization Strategies

Another noticeable field of helical stabilization using constrained peptide was achieved with *β*-peptides by groups of Seebach, Gellman, and DeGrado [60, 61]. The design of *β*-peptides that adopt helical conformations in organic solvent and water has been observed with conformationally restricted cyclic amino acids or side chain electrostatic interactions. *β*-peptide foldamers are composed of *β*-amino acid residues and are capable of adopting helical structures [62–64]. Foldamers are artificial nonpeptidic oligomers displaying protein-like function to target receptors whilst resisting proteolytic degradation [65]. Recent work has shown that heterogeneous backbone foldamers compared to homogeneous backbones ones have more benefits in designing stable conformation [66]. The Schepartz, Seebach, and Gellman groups have demonstrated the potentials of *β*-peptide foldamers to target proteins involved in transcription [65, 67, 68]. 

Last, synthetic agents have been shown to act as nucleators of helix formation. By providing rigidly constrained, appropriately placed hydrogen bonds to one end of a peptide, Kemp et al. [69, 70], Austin etal. [71], and Kazmierski et al. [72] have all provided a means for nucleation of helicity.

## 2. Stabilized and Destabilized ***β***-Sheets ****and ***β***-Turns

The mimicry of *β*-turns is a fertile area of research. Notable examples include the use of *meta*-amino-benzoic acid by DeGrado, the 9-membered macrocycles of Olsen and Kahn, 13-membered Ala-Gly turn analogues, and the macrocyclic *β*-turn mimetic of Ellman et al. [73, 74]. Burgess has reported several solid-phase syntheses of ring-fused C^10^ motifs and is now using these mimetics to function as synthetic antibody models [75]. Cochran and coworkers have recently outlined a minimal peptide scaffold for *β*-turn display employing disulphide-cyclized *β*-hairpins. They found that tryptophan in the 3 positions of a constrained *β*-hairpin of the form CX_8_C was found to confer stability to the turn conformation. Because the scaffold is composed of natural amino acids, it is amenable to the development of peptide libraries on phage having limited conformational diversity [76, 77].

**Figure 7:**
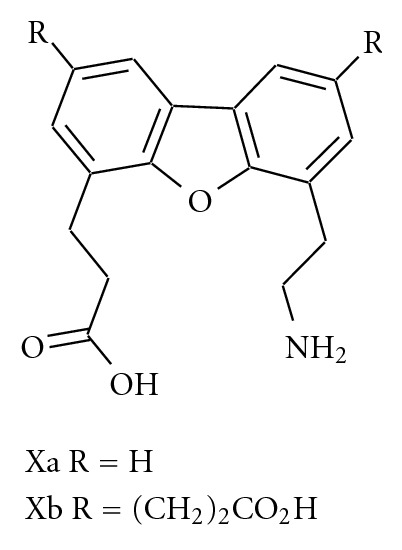
The dibenzofuran scaffold was employed as a synthetic *β*-turn mimetic.

Kelly et al. have developed *β*-turn mimetics-based dibenzofuran scaffold that induces *β*-hairpin formation in small peptides by replacing the *i* + 1 and *i* + 2 residues of a *β*-turn (Figure 7) [78–81]. It was shown that the incorporation of X into a WW domain of PIN1, a mitosis cell cycle regulator, can result in increased stability of the resultant miniprotein relative to the wild-type protein [82, 83]. The WW domain was named after two conserved tryptophan residues found in over 200 members in this protein family. This domain is readily accessible by solid-phase peptide synthesis (SPPS). Synthesized proteins exhibit cooperative folding transitions that facilitate kinetic and thermodynamic analysis. Such miniproteins represent useful probes of the role of the loop region with regard to the folding of *β*-sheets and the role of loops in general. The WW domain of PIN1 folds into a three-strand antiparallel *β*-sheet with two loops, which contains six and four residues, respectively. As loop 1 is solvent exposed and therefore not involved in other interactions, it was decided to incorporate a turn mimetic at this site. The protein exhibits what appears to be an unusual type II *β*-turn from S16 to S18 centered around R17 (*i* + 1) and S18 (*i* + 2). To test if this was important or not, S18 and S19 were substituted with known *β*-turn mimics dPro-Gly and Asn-Gly and the resultant mutants found to have little difference in structural stability. Incorporation of the dibenzofuran mutant resulted in a protein that was thermally less stable by 10°C. However, it was shown to be slightly more stable to chaotropic denaturation by 0.5 kcal/mol. Subsequent work has shown that acidic side groups tethered to the back side of the dibenzofuran allow incorporation of the turn mimetic with an increased stability comparable to that of the wild-type protein resulting from the improved solvation of the turn segment.

Arnold et al. have shown that incorporation of *β*-turn mimetics into a critical turn region of RNase A using expressed protein ligation (EPL) results in comparable stability and function to the wild-type enzyme [84]. In EPL, a modified intein is used to create a biosynthetic protein fragment containing a C-terminal thioester. The thiolate of an N-terminal cysteine residue in a synthetic peptide attacks the thioester to generate an amide bond within a semisynthetic protein. It is observed that the installed *β*-peptide module is not only tolerated by the protein structure but actually increases its stability by the measurement of ΔTm.

Whilst the design and synthesis of *β*-turn mimetics is a well-developed area, the stabilization and destabilization of *β*-sheet conformation lags behind. This stems from a less well-developed understanding of the factors that contribute to the stability of the *β*-sheet conformation, and in this regard the work of Searle et al. [85–87], Gellman [88], Schenck and Gellman [89], Fisk and Gellman [90], and others is advancing the field. Nowick et al., Schneider and Kelly, and others have shown that the use of appropriately designed templates can act as nucleation sites for *β*-sheets [91–96]. Norbornene has been shown to represent an effective nucleation template for *β*-sheets [97–99]. Cofacial positioning of amino and carboxy groups on a rigid aromatic scaffold allows the correct presentation of peptide strands necessary for antiparallel sheet formation, a strategy that has also been employed by Kelly to develop the turn mimetic discussed in the previous section. Nowick et al. have used 5-amino-2-methoxybenzoic acid hydrazide tethered to the upper or lower amines of a 1,2-diaminoterminated peptide strand [95]. The nonnatural amino acid modules serve as a *β*-strand mimic that can hydrogen-bond to peptide strands in an extended conformation. Recent studies have focused on the covalent linkage of two such units that they can hydrogen-bond to either sides of the extended form of a polypeptide forming a triply templated sheet. 

Finally, Zeng et al. have shown that nucleation of sheet structures can be achieved in organic solvents through the use of a DDAD : AADA hydrogen-bonded duplex to which are attached two peptide stands [100]. These results augur well for the development of stabilized *β*-sheet structures in aqueous media. Metal-ligand coordinative bonds have also been employed to great effect in the stabilization of *β*-sheets conformations. These include ruthenium-bipy coordination at an allosteric site, cobalt-bipy coordination at appropriately spaced sites within the peptide chain, cobalt-bipy coordination between appropriately spaced nonnatural amino acids, and various catechol-substituted peptides. To demonstrate these principles, an example from Schneider and Kelly has shown that 6,6′-bis(acyl amino)-2,2′ bipyridine substituted strands in the absence of metal ions are spaced far apart from each other due to pyridine-pyridine repulsion [96]. In the presence of copper (II), however, the pyridine nitrogen and the carboxyl functional groups coordinate with the metal, enforcing a linear square planar conformation from which a polypeptide chain is projected. The resulting antiparallel orientation of the two peptide chains is conducive to *β*-sheet formation. Likewise, 1,1′-bis-carboxy ferrocenes allow the projection of peptide side arms in a parallel orientation.

## 3. Regulating Protein-Protein Interactions ****with Constrained Peptides

**Figure 8:**
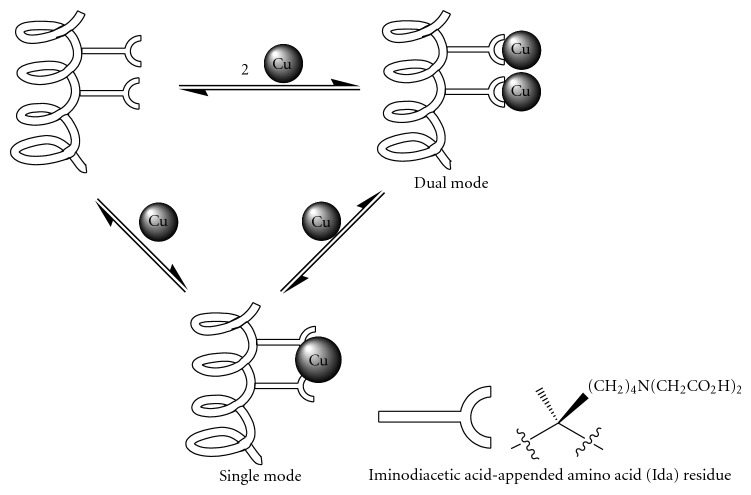
Iminodiacetic acid-appended amino acid (Ida) incorporated S-peptide of RNase S′ stabilizes upon binding to Cu(II) ion.

Incorporation of iminodiacetic acid-appended amino acid (Ida) residues into the S-peptide of RNase S′ allows regulation of the stability of the complex it formed with the S-protein and thus regulation of RNase S′ activity as shown by Hamachi et al. [36]. This elegant design involves installments of two Ida residues, which are strong chelators for transition metal cations in aqueous solution. Due to the hydrophilicity characteristics of the acid side chains, the peptide was oriented with the metal binding site exposed to the solvent side. It is shown that doubly replaced Ida in the S-peptide at the *i* and *i* + 3 sites cooperatively binds a Cu^2+^ cation then results in an increase of the helix content (Figure 8), which is called single-mode binding. This peptidocopper complex is able to pick up another Cu^2+^ if the concentration of Cu^2+^ is high enough to form a dual-mode complex. The single mode with stoichiometry of 2 : 1 binding enhanced the activity of A6/E9Ida-Rnase S′, while the dual mode with 2 : 2 stoichiometry suppressed it. The opposite effects from different binding modes provided a switch to control the enzyme activities by addition of chemical reagents. Thus, it has to be a very promising frontier work of molecular level engineering.

C5a is believed to be a pathogenic factor in a range of immunoinflammatory diseases including sepsis and may therefore serve as a useful target for anti-inflammatory agents. Finch and coworkers produced cyclic peptides that mimic the structure of active peptide inhibitors of the C5a receptor. These were derived from peptide fragments containing the binding region obtained by site-directed mutation of C5a [101]. The most potent of these inhibitors bound to the receptor with IC_50_ = 0.3 M as measured in a competition assay using ^125^I-labeled C5a. The cyclic peptide displayed antagonist potency in the presence of 100 nM C5a with IC_50_ = 20 nM as measured by myeloperoxidase release from cytochalasin B stimulated human polymorphonuclear cells. Compared to control animals, anesthetized rats dosed intravenously with X when subjected to either C5a or lipopolysaccharide (which stimulates an increase in endogenous C5a) displayed significantly reduced neutropenia (decrease in circulating PMNs) and blocked the elevation of serum TNF-*α* and IL6, two proinflammatory cytokines [102].

A delightful example employing constrained peptides that act as inhibitors of protein-protein interactions has been described in the work reported by Garcia-Echeverria et al. [103]. Antibodies were used to identify the binding region between p53 and human double minute 2 (HMD2). Synthetic peptides from the N-terminus of p53 were then used to probe further the binding region on HDM2. A hexapeptide comprising residues 18–23 of p53 was identified as the minimum binding epitope for HDM2 recognition with an IC_50_ = 700 *μ*M. However, a peptide comprising 12 residues from p53 displayed a more respectable IC_50_ = 8.7 *μ*M and was used as a starting point for further studies. Phage display identified a 12-mer with 28-fold improved potency, and then synthetic truncated peptides were used to determine a minimum length required for micromolar affinity towards HDM2 of eight amino acids. X-ray crystallographic and NMR spectroscopic data proved indispensable to the further optimization of the 8-mer. Crystallography revealed that a 15-mer p53-derived peptide bound in a deep hydrophobic cleft on HDM2 and adopted a helical conformation. This also identified relevant contacts with the HDM2 protein and residues suitable for structural biasing. This was confirmed further with solution-based studies. A helical conformation was promoted (thus decreasing the entropic cost of binding) by introduction of *α*,*α*-disubstituted amino acids at noncritical residues for interaction with HDM2. A tyrosine residue was also replaced by phosphonomethylphenylalanine to introduce an electrostatic interaction with Lys-94 in HDM2, and substitution on the tryptophan residue was included to better complement a hydrophobic “hole” in the HDM2 protein. These combined modifications resulted in a peptide that inhibited binding of p53 to HDM2-GST with IC_50_ = 5 nM for representing a 1700-fold improvement in overall binding affinity. 

Many groups have succeeded in developing diverse stabilized helices and helix mimetics to target the interaction between p53 and HDM2, including terphenyl-based helical mimetics by Yin et al. [104], hydrocarbon stabilized helical peptide by Bernal et al. [23], *β*-hairpin protein epitope mimetics by Fasan et al. [105], helical *β*-peptide inhibitors by Kritzer et al. [106] and Murray and Gellman [107], and oligobenzamide proteomimetic inhibitors by Plante et al. [108]. Shaginian and coworkers also design an approach utilizing solution-phase synthesis to set up an *α*-helix mimetic library for screening of the protein-protein interaction inhibitors [109].

Several miniproteins have been designed to target the interaction between the activation domain of p53 and HDM2, including the superTIP (thioredoxin insert protein) by Böttger et al. [110], and helical scaffolds derived from scorpion toxin and apamin-derived stingins by Lu et al. [111, 112]. Kritzer et al. utilized the “grafting” technology in combination with a functional selection to mature the miniprotein ligands for globular protein receptor rapidly [113]. This group obtained cell permeable *β*-peptide mimicking p53 activation domain to inhibit the p53/HDM2 complex formation [114].

**Figure 9:**
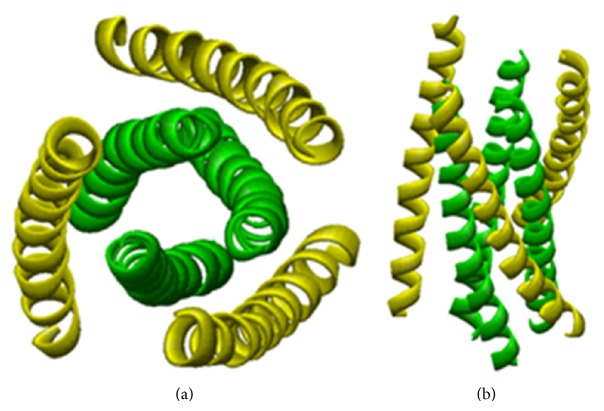
(a) Top and (b) side views of the crystal structure of the fusion active core of gp41. The helices correspond to the N36 (green) and the C34 peptide (yellow).

A further example of the enhanced biological effects that constrained peptides exert upon their targets involves peptide inhibitors of the envelope glycoprotein of HIV-1 (gp41), which mediates membrane fusion between the virus and target cells. This hexameric domain contains an N-terminal glycine-rich fusion sequence and two helical regions containing hydrophobic 4-3 heptad repeats denoted as N- and C-helical regions. The N-terminal portion forms a parallel trimer, and the C-terminal portion surrounds this inner core as indicated by X-ray crystallography (Figure 9) [115, 116]. Upon binding cell surface receptor, gp41 undergoes a conformational change that exposes the hydrophobic N-helical regions and allows the fusion peptides to insert into the host cell membrane. Inhibitors of HIV-1 entry into host cells could be envisioned by binding the N-terminal core of the coiled coil trimer before the C-terminal peptide binds to form the gp41 fusion protein. The hydrophobic pocket region of HIV-1 gp41 is an attractive target, because it may be less prone to drug-resistant mutations [115, 117–119]. 

Earlier work has demonstrated that the C-terminal portion of gp41 was a potent inhibitor of viral membrane fusion [120, 121]. Fragments of the C-terminal peptide of gp41 (residues 643–678) were designed to contain covalent diaminoalkyl tethers between glutamate *i*, *i* + 7 residues. Peptides having one or two covalent tethers were shown to be significantly *α*-helical from 7°C to 37°C by circular dichroism. ELISA quantified the inhibition of viral infectivity from the amount of p24 antigen (from cell lysates) found in cells treated with either free HIV virus or HIV virus particles incubated with inhibitor peptides bearing one or two cross-linked glutamines. Peptides that were cross-linked on the face of the peptide proposed to bind the core trimer were inactive, whilst peptides with two cross-links were more effective than those with one. Though in these studies, short C-peptides corresponding to the pocket-binding region failed to inhibit HIV-1 entry, most likely due to weak binding to the target. 

A group from Genentech reported inhibition of HIV-1 infectivity by constrained *α*-helical peptides [122]. Because short peptides generally do not form stable *α*-helices in solution, a covalent crosslink was used to link the *i* and *i* + 7 amino acid residues and forced the peptide to adopt the helical presentation. The induced helicity was confirmed by CD analysis. HIV 24, HIV 30, and HIV 31 showed characteristic CD pattern of *α*-helices, while unconstrained peptide HIV 35 has an almost featureless spectrum. Then the constrained peptides were tested in viral infectivity assays. HIV35, in which the C-terminus of DP178 was chopped, showed a dramatic drop in activity compared to the full length of the helical region of gp41. The singly constrained peptide, HIV 24, partially restored the activity. By contrast, the doubly constrained peptide showed comparable affinity to DP178, suggesting a correlation between helicity and inhibitory potency. The lack of inhibition by HIV30 showed the exposed face of the helix was required.

**Figure 10:**
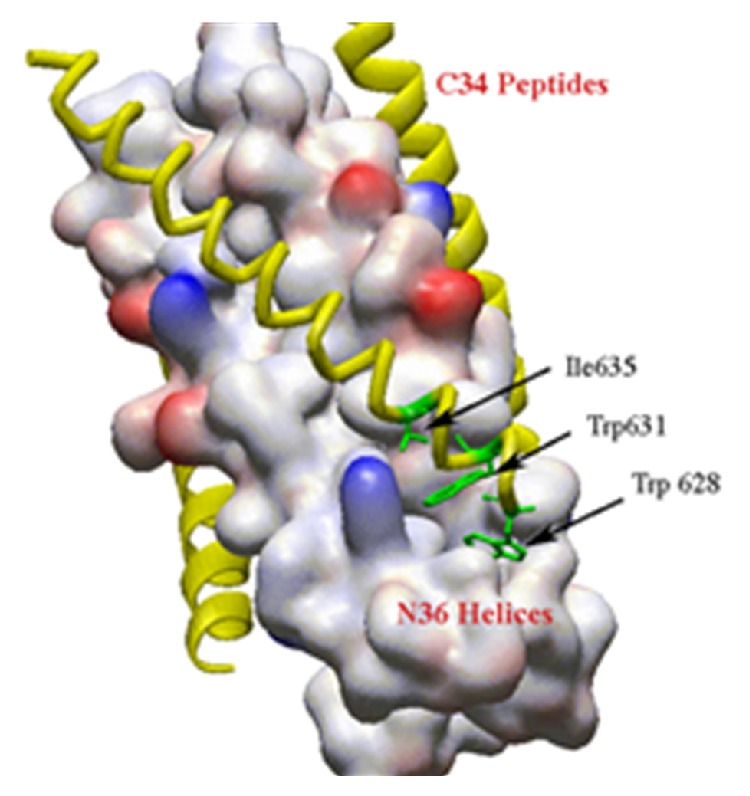
A surface model of the trimeric coiled coil. The interacting side chains at *i*, *i* + 3, *i* + 7 positions of C14 peptide are shown in green.

Sia and coworkers have also shown that constrained C-peptides disrupt the assembly of the hexameric gp41 core that leads to HIV-1 viral fusion with host cells [123]. The two strategies that were employed to stabilize the helix are (1) the use of unnatural helix-favoring amino acids and (2) covalent cross-linkers. *α*-Aminioisobutyric acid (Aib) was a well-studied unnatural amino acid that contains two methyl groups attached to the a carbon and its *α*,*α*-disubstituted structure restricts its conformation to 3_10_ helices so substitution of Aib into a peptide sequence can dramatically increase its helical propensity. As an alternative helix-stabilizing strategy, *α*,*ω*-diaminoalkane was used to connect two glutamic acid residues at *i*, *i* + 7 positions in order to induce the helicity. Both of strategies succeeded in promoting the inhibitory activities of a C14 peptide that targets the HIV-1 gp41 hydrophobic pocket. C14Aib and C14linkmid, respectively, showed 144 *μ*M and 35 *μ*M IC_50_ in the cell-cell fusion assay, compared with over 500 *μ*M IC_50_ of the wild-type C14 peptide. C14linkmid also shows measurable inhibitory activity in a luciferase-based viral infectivity assay at an IC_50_ approximately 500 *μ*M. A good correlation between binding affinity to the gp41 hydrophobic pocket and cell-cell fusion inhibitory activity was observed. Various structural analyses using NMR spectroscopy and X-ray crystallography were carried out to confirm the constrained peptide bind to the hydrophobic pocket on the surface of HIV-1 gp41. It was revealed that the C14linkmid binds to the HIV-1 gp41 hydrophobic pocket in essentially the same conformation as the pocket-binding region of a linear C-peptide (Figure 10). Furthermore, the spatial arrangements of the side chains in the hydrophobic pocket (Trp-628, Trp-631, Ile-635, Leu-568, Trp-571) are virtually identical in the two structures, suggesting very similar interacting modes. Though the two most potent constrained peptides, C14linkmid and C14Aib, did not adopt significant helical content shown by CD spectroscopy. It is perhaps due to that the helical conformation of highly constrained peptide was not favored in the bound complex.

Human parathyroid hormone (hPTH) binds to certain receptors on the gastrointestinal tract, the kidney, and the bone issue, regulating calcium homeostasis in the body. Several groups reported approaches of using constrained peptide to obtain helical conformation and thus enhanced proteins to target hPTH or hPTHrP. Barbier et al. showed that an extended helical region of hPTH was stabilized by an *i*, *i* + 4 side chain-to-side chain amide bridge between a basic lysine residue and an acidic aspartate or glutamate residue [124]. Adenylyl cyclase activities were measured to decide the constrained peptides' affinity to interact with hPTH receptor. Peptide **2** with a constraining side chain linking the positions of 22 and 26 showed bioactivities 6-fold stronger than the linear peptide. Introduction of a second constraint leads to peptide **4** with an elevated EC_50_ of 0.13 nM. Condon and coworkers further developed the idea with addition of the third lactam constraint. Their binding assay which used the recombinant hPTH/hPTHrP receptor showed the tricyclopeptide **5** could reach the similar potency with peptide **4** at the low nanomolar level [125]. Peggion and coworkers used to take another strategy to stabilize the helices of bovine parathyroid hormone [126]. C*α*-tetrasubstituted amino acids were used to induce the formation of 3_10_ helices. Structure-function relationships were studied with several analogues with an introduced Aib residue at the positions 12 and 13 (Table 1). It was observed that the analogue **7** has the best affinity with an EC_50_ of 0.2 nM.

Most recently, another example to use Aib constraint in the designed peptide to induce helical conformation was presented by Das et al. lately [127]. The *α*-aminoisobutyric acid-(Aib-) rich peptides were well documented to form 3_10_ helices due to the steric strain on the C*α*-position. The targeted 3_10_ helices regions are in general shorter than the *α*-helices so they made the constrained peptide a very attractive strategy to adopt the secondary conformation [128]. In the approach to inhibit *γ*-secretase, which is a critical player in the process of development of Alzheimer's disease, Wolfe and coworkers designed short peptides sequence ranging from six to ten residues (two or three of them are Aib residues) based on the helical region APP transmembrane domain. CD experiments were conducted to study the conformation, and characteristic spectra were observed. Though further clarification of the helical conformation was not shown, presumably because the spectroscopic differentiation of the 3_10_-helix from *α*-helices is traditionally difficult. Nonetheless, low micromolar level IC_50_ was observed in the purified enzyme assay. Further information provided that the adopted conformation played an important role in the inhibition, and partial inversion of the *α*-stereocenters led to a 100-fold loss of potency, suggesting that the helical conformation rather than hydrophobicity is critical in the inhibition of *γ*-secretase.

Schepartz's group developed a general solution called “protein grafting,” which, often used in combination with molecular evolution, identified miniature protein with high affinity and specificity for proteinaceous and nucleic acid targets [129–133]. A recent application of this approach is the identification of the high-affinity ligands for the CBP KIX domain [134]. The complex between the KIX domain of the transcriptional coactivator protein (CREB binding protein, CBP) and the kinase-inducible activation domain (KID) of the transcription factor CREB is a challenging target to recognize as the KID-binding cleft on the surface of KIX is shallow and more resembles the solvent-exposed protein surface than a typical *α*-helical-binding groove [135]. Hydrophobic interaction contributes significantly to the free energy of KID^P^ · KIX complex formation: the side chains at *i*, *i* + 3, *i* + 4, *i* + 7 positions (Try134, Ile137, Leu138, Leu141) on the same face of the helical region of CREB KID are interacting with the surface of KIX. These residues are grafted onto the solvent-exposed *α*-helical face of the small yet stable protein avian pancreatic polypeptide (aPP) [136, 137]. The resulted phosphopeptide PPKID4^P^, with the additional functional epitope (G**P**S**QP**T**YP**GDDAPVRRLSFFYILLDLYLDAPGVC) to recognize CBP KIX surface on its desired secondary conformations, exhibited high affinity (*K*_*d*_ = 562 ± 41 nM) and high selectivity over carbonic anhydrase (*K*_*d*_CA = 106 ± 12 *μ*M) and calmodulin (*K*_*d*_CalM = 52 ± 12 *μ*M) that also bind hydrophobic or *α*-helical ligands [4, 138, 139]. It is particularly noteworthy that the grafted miniature protein showed high preference (100-fold) to recognize CBP KIX over calmodulin, whose native ligand smooth muscle myosin light-chain kinase (smMLCK) also adopts an *α*-helical conformation with the key binding residues at *i*, *i* + 3, *i* + 7 positions (Trp800, Thr803, and Val807) [139], indicating the “protein grafting” is a sensitive method to identify inhibitors of protein-protein interactions while further structural affirmation of the inhibition mode is desirable.

**Figure 11:**
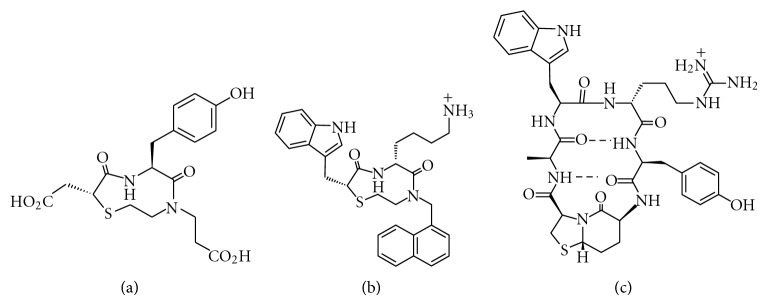
Structure of some *β*-turn surface mimetics.

Ellman and coworkers developed a general method to prepare constrained peptidyl mimetics of *β*-turn based on solid-phase synthesis with a variety of side chains functionality at the *i* + 1, *i* + 2, and *i* + 3 positions incorporated [73, 74]. As an application of this design, a library of 2302 small molecule *β*-turn mimetics generated from the solid-phase synthesis was screened for inhibition of *α*4*β*1 intetrin-CS1 splice variant binding interaction [140]. Active compounds were identified with the best lead (Figure 11(a)) with an IC_50_ of 5 *μ*M. In another search of potential to target human somatostatin receptors (hSST), a focused library of *β*-turn mimetics based upon the crucial Trp-Lys motif found in the turn region of somatostatin was screened and resulted in the identification of a potent heterocyclic ligand (Figure 11(b)) with an IC_50_ value of 87 nM against subtype 5 human somatostatin receptor (hSST_5_) [141]. With the aid of the vector search program CAVEAT [142], Etzkorn and coworkers designed a chimeric cyclic peptide (Figure 11(b)) to structurally mimic a *β*-turn region of tendamistat, which is a 74-residue proteinaceous inhibitor of *α*-amylase (Figure 11(c)) [143]. The targeted area of tendamistat is the ^18^Trp-^19^Arg-^10^Tyr residues that occupy the *i* + 1 to *i* + 3 positions of a slightly distorted type I *β*-turn, with the ^19^Arg side chain sandwiched between the adjacent aromatic rings. In order to evaluate the abilities of the chimeric peptides to inhibit *α*-amylase, an assay was developed on the basis of the hydrolysis of *p*-nitrophenyl maltotrioside (*p*-NPG_3_). Moderate affinities of the cyclic hexapeptides mimetics were observed with Ki values of 14–32 *μ*M, while tendamistat inhibits *α*-amylase with a Ki of 0.2 nM.

CBP and the related protein p300 are transcriptional coactivators implicated in cell growth, differentiation, and embryonic development. Recent work of Henchey et al. demonstrated that HBS helices mimicking a helical segment in the C-terminal activation domain of hypoxia-inducible transcription factor (HIF)-1*α* can bind to the cysteine-histidine rich 1 (CH1) region of CBP/p300 and inhibit transcription of hypoxia-inducible genes in cell culture [30]. Hypoxia-inducible genes encode vascular endothelial growth factor (VEGF) and its receptor VEGFR2 involved in the induction of solid tumors angiogenesis (growth of new blood vessels) [144]. The ligands inhibiting hypoxia-inducible gene expression may be designed for the treatment of neovascularization in solid tumors. 

## 4. Conclusions

In this paper, we have highlighted the progress made in the stabilization of protein secondary structures. These peptidomimetics render novel strategies to disrupt therapeutically important protein-protein interactions such as the gp41 complex formation, the p53-HDM2 interactions, and Bcl-Bak family proteins that play important roles in apoptosis. Constrained peptides, as miniature protein mimetics, present the essential recognition functionality of secondary structures involved in protein-protein interactions, providing a generally applicable method to target the “undruggable” protein-protein interactions. It is also possible to move beyond secondary structure and disrupt protein-protein interactions mediated by interfacial contact of large surface areas.

## Figures and Tables

**Table 1:** Sequences of constrained human parathyroid hormone peptides.

Peptide	EC_50_ (nM)
hPTH-(1-31)NH_2_ (**1**)	19.9
[Leu^27^]cyclo(Glu^22^-Lys^26^)-hPTH-(1-31)-NH_2_ (**2**)	3.3
cyclo(Lys^18^-Asp^2^2)[Ala^1^, Nle^8^, Lys^18^, Asp^22^, Leu^27^]hPTH(1-31)NH_2 _(**3**)	0.29
bicyclo(Lys^18^-Asp^22^, Lys^26^-Asp^30^)[Ala^1^, Nle^8^, Lys^18^, Asp^22^, Leu^27^]hPTH(1-31)NH_2_ (**4**)	0.13
tricyclo(Lys^13^-Asp^17^, Lys^18^-Asp^22^, Lys^26^-Asp^30^)[Ala^1^, Nle^8^, Lys^18^, Asp^17, 22^, Leu^27^]hPTH(1-31)NH_2 _(**5**)	0.14
[Nle^8, 18^, Nal^23^, Tyr^34^]bPTH(1-34)-NH_2_ (**6**)	0.85
[Nle^8, 18^, Aib^12, 13^, Nal^23^, Tyr^34^]bPTH(1-34)-NH^2^ (**7**)	0.2
